# Magneto-Photochemically Responsive Liquid Crystal Elastomer for Underwater Actuation

**DOI:** 10.1021/acsami.4c14704

**Published:** 2025-01-09

**Authors:** Yasaman Nemati, Qi Yang, Fereshteh Sohrabi, Jaakko V. I. Timonen, Carlos Sánchez-Somolinos, Mari Honkanen, Hao Zeng, Arri Priimagi

**Affiliations:** † Faculty of Engineering and Natural Sciences, Tampere University, P.O. Box 541, FI-33101 Tampere, Finland; ‡ Qingdao University of Science & Technology, Qingdao 266042, China; § Department of Applied Physics, Aalto University School of Science, Puumiehenkuja, 202150 Espoo, Finland; ∥ Instituto de Nanociencia y Materiales de Aragón (INMA), CSIC-Universidad de Zaragoza, Departamento de Física de la Materia Condensada, Zaragoza 50009, Spain; ⊥ Centro de Investigación Biomédica en Red de Bioingeniería, Biomateriales y Nanomedicina (CIBER-BBN), Instituto de Salud Carlos III, Madrid 28029, Spain; # Tampere Microscopy Center, Tampere University, P.O. Box 692, 33014 Tampere, Finland

**Keywords:** liquid crystal elastomer, shape morphing, magnetoresponsive, azobenzene, soft actuator

## Abstract

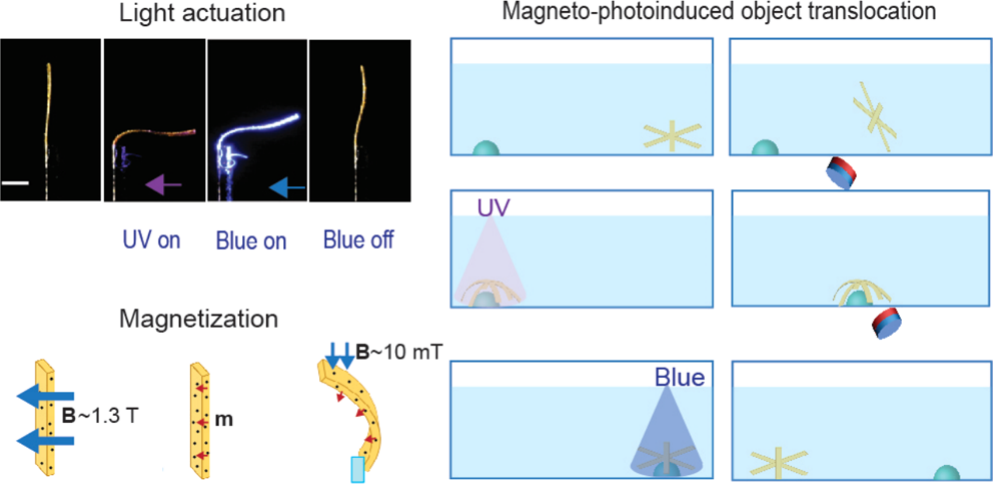

The quest for small-scale, remotely controlled soft robots has led to the exploration of magnetic and optical fields for inducing shape morphing in soft materials. Magnetic stimulus excels when navigation in confined or optically opaque environments is required. Optical stimulus, in turn, boasts superior spatial precision and individual control over multiple objects. Herein, we bring these two methodologies together and present a monolithic liquid crystal elastomer (LCE) system that synergistically combines magnetic and photochemical actuation schemes. The resultant composite material showcases versatile possibilities for underwater actuation, and we demonstrate robotic functionalities where the optical and magnetic response can be leveraged in different tasks (object gripping and object translocation, respectively) or where light can be used as a control signal to tune the magnetically induced actuation. Combining these two remote actuation methods offers powerful, dual-mode control in wireless, small-scale robotics, especially in submersed environments due to their isothermal nature.

## Introduction

Materials that exhibit dynamic shape changes in response to external stimuli are pivotal in the advancement of small-scale soft robotics.^1−3^ Among these, liquid crystal elastomers^4^ (LCEs) are particularly notable due to their ability to undergo large and reversible deformations.^5,6^ LCEs combine the mechanical properties of loosely cross-linked polymer networks with the anisotropic properties of liquid crystals, enabling, e.g., complex shape changes via molecular alignment engineering,^7,8^ in response to a wide variety of stimuli.^9−13^ Additionally, reprogrammability can be incorporated into LCEs via dynamic covalent bonds.^14−17^ These features pave the way for remote actuation and locomotion in diverse environments, i.e., on land,^18−20^ in air,^21−23^ and underwater.^24−26^

The predominant remote actuation methods in stimuli-responsive materials are magnetic and light-driven actuation. Magnetic actuation uses external magnetic fields to generate forces or torques, enabling the remote manipulation of robotic structures.^27−29^ This approach is particularly effective in opaque environments, such as within the human body, making it potential for noninvasive medical procedures and remote exploration. In addition to magnetic torque, the magnetothermal effect, where high-frequency magnetic fields induce heat through Eddy currents^30^ or magnetic hysteresis,^31^ represents another dimension of magnetically induced actuation and shape changes. As drawbacks, magnetic systems often face challenges related to the complexity of the required hardware, limited operational distances, and difficulties in controlling multiple objects independently.^32^ Examples of magnetically actuated LCEs are scarce,^11,33−35^ light being the predominant trigger for remote actuation. Light-driven actuation relies on either photochemical^36^ or photothermal^37^ effects that disrupt the anisotropic molecular alignment of LCEs, causing stress/strain gradients and macroscopic shape changes. Light provides precise spatiotemporal control and localized actuation capability.^38^ However, the effectiveness of both the photochemical and photothermal approaches is constrained by the need for a clear line of sight and limited light penetration depth. This highlights the need for a hybrid approach that combines the strengths of both magnetic and light actuation while minimizing their respective drawbacks.^32^

Underwater remote actuation of LCEs is of particular interest due to its important applications in microfluidics and biomedicine, as well as its capability to emulate the motions and functionalities of biological systems.^39^ LCEs can mimic biological microswimmers,^4,40^ ephyra,^26^ and artificial aquatic cilia.^41,42^ Examples range from underwater robotic operation and bioinspired propulsion to intricate studies of the hydrodynamic mechanism of soft-bodied organisms. In this context, the combination of magnetic and light stimuli can be particularly attractive as these two stimuli can be harnessed to conduct different tasks within the multiresponsive LCE construct.^34,43^ It is noteworthy that albeit some examples exist,^4,24,44−46^ photothermal actuation mechanism is notoriously inefficient in aqueous conditions due to high thermal dissipation. Photochemical actuation, in turn, utilizes reversible *trans–cis* photoisomerization of, e.g., azobenzene molecules in response to ultraviolet/visible (UV/vis) light to disrupt/restore the molecular alignment.^47^ The resultant macroscopic shape changes take place (almost) isothermally, rendering the photochemical actuation mechanism much more attractive for underwater applications, especially in the context of biomedicine.

The integration of magnetic particles into LCEs has been recently explored to enhance actuation dynamics, showing programmable deformations. However, these studies have typically combined magnetic actuation with photothermal/thermal actuation to achieve dual responsiveness through layered or bilayer structures.^11,31,33−35,48^ While effective under certain conditions, photothermal actuation faces limitations in thermally sensitive or aqueous environments due to heat dissipation and temperature fluctuations. A notable exception is an artificial aquatic “polyp” that combines a magnetically driven base and a photochemically controlled gripper to wirelessly attract, grasp, and release objects, yet comprising two separate structural components for the magnetic and light-driven actuation.^49^ The integration of magnetic and photochemical actuation within a single monolithic LCE system remains unexplored.

Herein, we address this gap by developing a dual-responsive LCE system that combines the robustness of magnetic actuation with the precision of the photochemical response facilitated by azobenzene derivatives in a single, monolithic matrix. Through this combination, we create a design with dual actuation capability while retaining simplicity and reliability across varied applications, particularly in underwater environments where photochemical actuation is advantageous. The system demonstrates effective integration of these two actuation modes, allowing for sufficient light penetration to maintain the efficacy of photochemical actuation even in the presence of hard magnetic microparticles at a relatively high mass ratio. The system is utilized in underwater object manipulation where magnetic fields are used to position the actuator and move objects that are gripped and handled via photochemical stimulus and in the optical reconfiguration of magnetically synchronized cilia, where photochemical stimulus is used to fine-tune the oscillatory behavior of the cilia in response to oscillating magnetic fields.

## Results and Discussion

**Figure 1:**
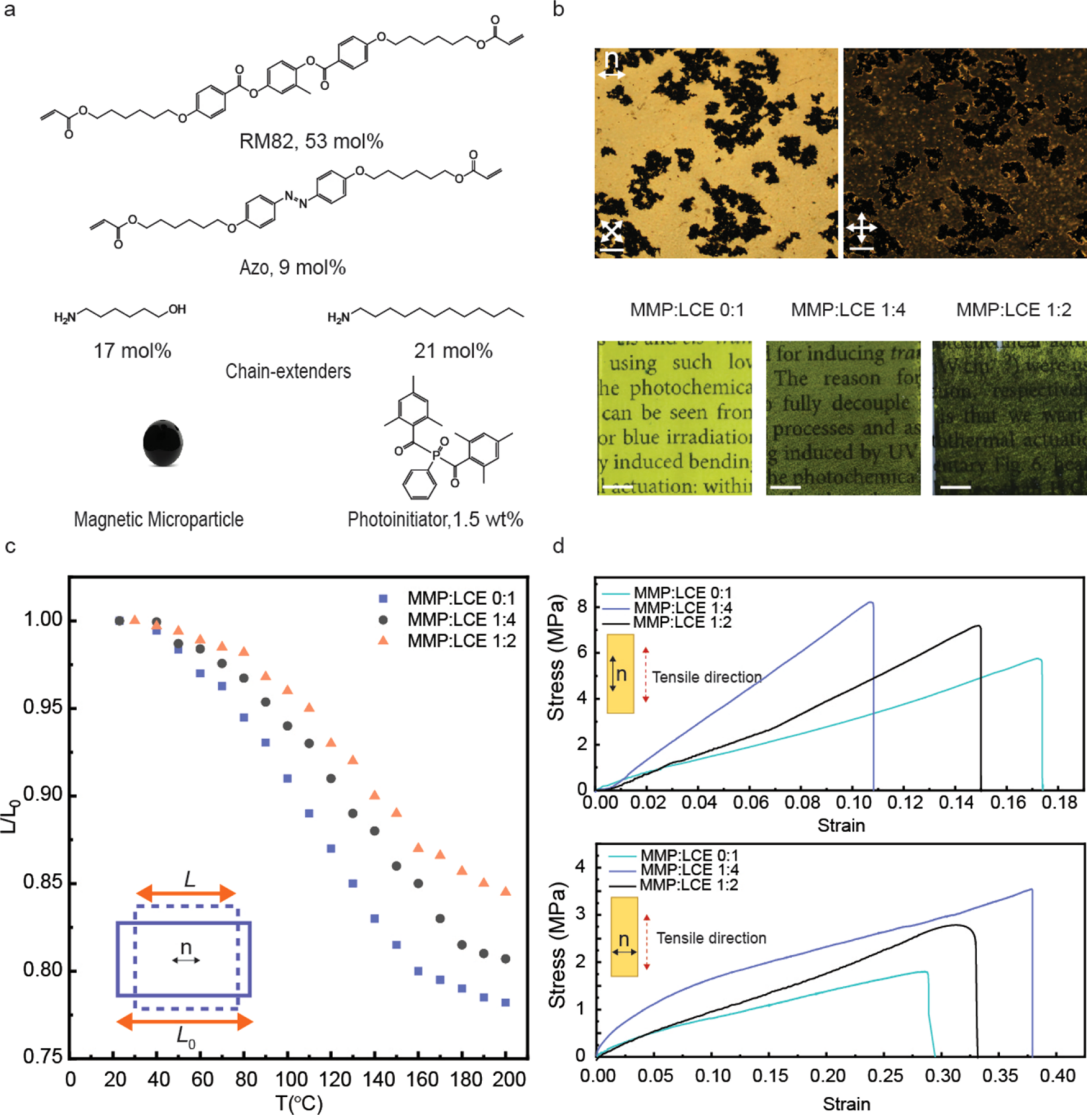
Material characterization. (a) Chemical structures of molecules utilized in the fabrication of magneto-photoresponsive LCE. (b) Top: Polarized optical microscopic images of the LCE–MMP composite (ρ = 1:4) at 0 and 45° angles between the molecular director and the polarizer. Bottom: Photographs of prepared films with varying MMP:LCE ratios. Scale bars: 200 μm (top), 5 mm (bottom). (c) Uniaxial contraction during heating from room temperature to 200 °C. Inset: A schematic representation of the deformation of a planar-aligned LCE, where *L*_0_ represents the original length, *L* is the contracted length, and ***n*** is the director orientation. (d) Stress–strain curves of the planar LCE–MMP composites with varying MMP/LCE ratios, parallel (top) and perpendicular (bottom) to the director orientation.

To prepare the magneto-photoresponsive LCEs, we first employed a chain extension method to oligomerize liquid crystalline diacrylate monomers and primary amines through aza-Michael addition reaction and then photopolymerized the remaining diacrylate end groups under UV irradiation to yield a loosely cross-linked polymer network.^8,50^ The photochemical responsivity is achieved by embedding photoisomerizable azobenzene diacrylates into the network. The magneto-responsivity is brought about by incorporating hard magnetic NdFeB microparticles (MMP, average diameter of 5 μm) into the polymerizable mixture. These particles were chosen for their high remanence and coercivity, allowing later magnetization of the LCE samples which yields subsequent strong responsiveness to weak magnetic fields.^51^ The synthesis process was carried out within surface-aligned cells with a thickness of about 50 μm to yield planar-aligned LCE strips. Further details on sample preparation can be found in Materials and Methods Section. All of the molecules used in this study are depicted in Figure 1a.

Incorporating MMPs into photochemically driven LCEs seems counterintuitive at first. Photoisomerization, as well as efficient curing of the photopolymerizable mixture, relies on sufficient light penetration into the film, while the MMPs absorb and scatter light over the whole UV–vis wavelength range. However, our observations suggest that the MMPs are not uniformly distributed throughout the LCE matrix, as evidenced by the formation of distinct regions with varying optical properties (Figure 1b, top). This heterogeneous distribution may act in our favor, allowing light penetration and efficient photoisomerization in the MMP-deficient regions. Despite the initial concerns, also curing appears to proceed even in the MMP-rich areas, possibly facilitated by the scattering properties of the MMPs, which could enhance light distribution within the material. Polarized optical microscopy confirmed that the LCE retained a planar director orientation even with the inclusion of MMPs (Figure 1b, top).

Figure 1b (bottom) presents a comparative visualization of LCE films with MMP:LCE mass ratios, denoted as ρ, of 0:1, 1:4, and 1:2. As ρ increases, the LCE gets darker and the optical scattering increases. However, the order parameter of the LCE, estimated via polarized absorption of the n−π* band of the azobenzenes, was found to be 0.54 (ρ = 0:1), 0.54 (ρ = 1:4), and 0.49 (ρ = 1:2), indicating that there is no notable disruption in the molecular alignment upon the inclusion of the MMPs at least up to a ratio of 1:4, and only a very minor decrease even at ρ = 1:2. This could be explained by the above-mentioned phase segregation, where the MMPs cluster into distinct regions within the LCE matrix, thereby leaving large areas of the composite system relatively undisturbed and maintaining good overall alignment. Further details on the order parameter determination are given in Note S1 and Figure S1. To further investigate the microstructure of the LCE-MMP composites, cross-sections of the samples with different ratios were characterized by analytical scanning electron microscopy (SEM; Figure S2). The SEM images reveal that the magnetic microparticles are distributed throughout the LCE matrix, with some clustering observed. Despite these clusters, the structural integrity of the LCE matrix is maintained, enabling reliable dual-responsive behavior. Elemental mapping of iron (Fe–K) confirms the presence and distribution of the magnetic microparticles within the matrix.

The planar-aligned, MMP-containing LCEs exhibit thermally induced contraction along the director, as illustrated in Figure 1c. The addition of MMPs systematically decreases the degree of contraction of the pure LCE, from 22 to 19% (ρ = 1:4) and 15% (ρ = 1:2) at a temperature of 190 °C. Based on the combination of higher transparency (Figure 1b), undisturbed order parameter, and higher thermal contraction along the director axis (Figure 1c), we decided to omit the 1:2 MMP:LCE ratio from further experiments and proceeded with ρ = 1:4, unless otherwise stated.

We next explored the azobenzene isomerization within the LCE-MMP composite. Working with relatively thick samples (50 μm), the absorbance of the π–π* band at near-UV was too high to be measured, and we used the *n*–π* transition at ca. 450 nm to monitor the reversible photoisomerization. Figure S3a presents the absorption spectra (400–750 nm) of (i) the pristine LCE-MMP composite, (ii) after irradiation with UV light (365 nm, 90 mW cm^–2^), and (iii) after subsequent irradiation with blue light (460, 120 mW cm^–2^). The intensification of the *n*–π* band upon UV irradiation and the retainment of the original state after irradiation with blue light are signatures of reversible *trans–cis* isomerization. As depicted in Figure S3b, the *cis*-lifetime, determined by single-exponential fitting of the thermal isomerization kinetics, is almost unchanged by the incorporation of MMPs, the lifetimes being 595 min (ρ = 0:1) and 583 min (ρ = 1:4). This highlights the retention of photochemical functionality despite the integration of the strongly absorbing magnetic particles. Irradiation with blue light leads to the isomerization of the *cis*-isomers back to the thermodynamically stable *trans-*form. The reversibility of the photoisomerization was demonstrated by a sequence of ten switching cycles (Figure S3c).

The incorporation of MMPs also modifies the mechanical properties of the LCE, as depicted by the strain–strain curves shown in Figure 1d. Parallel to the LC director, Young’s modulus of the material increased from 32 MPa in the pristine LCE without MMPs (ρ = 0:1) to 78 MPa in the LCE-MMP composite (ρ = 1:4). Perpendicular to the director, the corresponding values were 10 and 19 MPa, for the particle-free LCE and ρ = 1:4 LCE-MMP composite, respectively. This increase in stiffness highlights the role of the MMPs in reinforcing the polymer network. However, the fracture strain parallel to the director is reduced by the addition of MMPs, suggesting reduced deformability of the elastomer matrix. Although higher MMP content (ρ = 1:2) might be expected to further enhance the stiffness, the observed properties (Young’s modulus of 38 and 11 MPa parallel and perpendicular to the LC director, respectively) suggest that high concentration of black MMPs interferes with the polymerization process, influencing the mechanical performance. The differential scanning calorimetry (DSC) curves for the LCE–MMP composites with varying MMP:LCE ratios (0:1, 1:4, and 1:2) are shown in Figure S4. Only minor changes can be observed in the glass-transition temperature (*T*_g_; ca. −10, −7.5, and −10.5 °C for 0:1, 1:4, and 1:2 ratios), indicating that the incorporation of magnetic microparticles does not significantly alter the polymer properties. This observation aligns with previous studies,^31^ which reported that varying MMP concentrations in LCE composites had minimal impact on the nematic-to-isotropic phase transition temperature.

**Figure 2:**
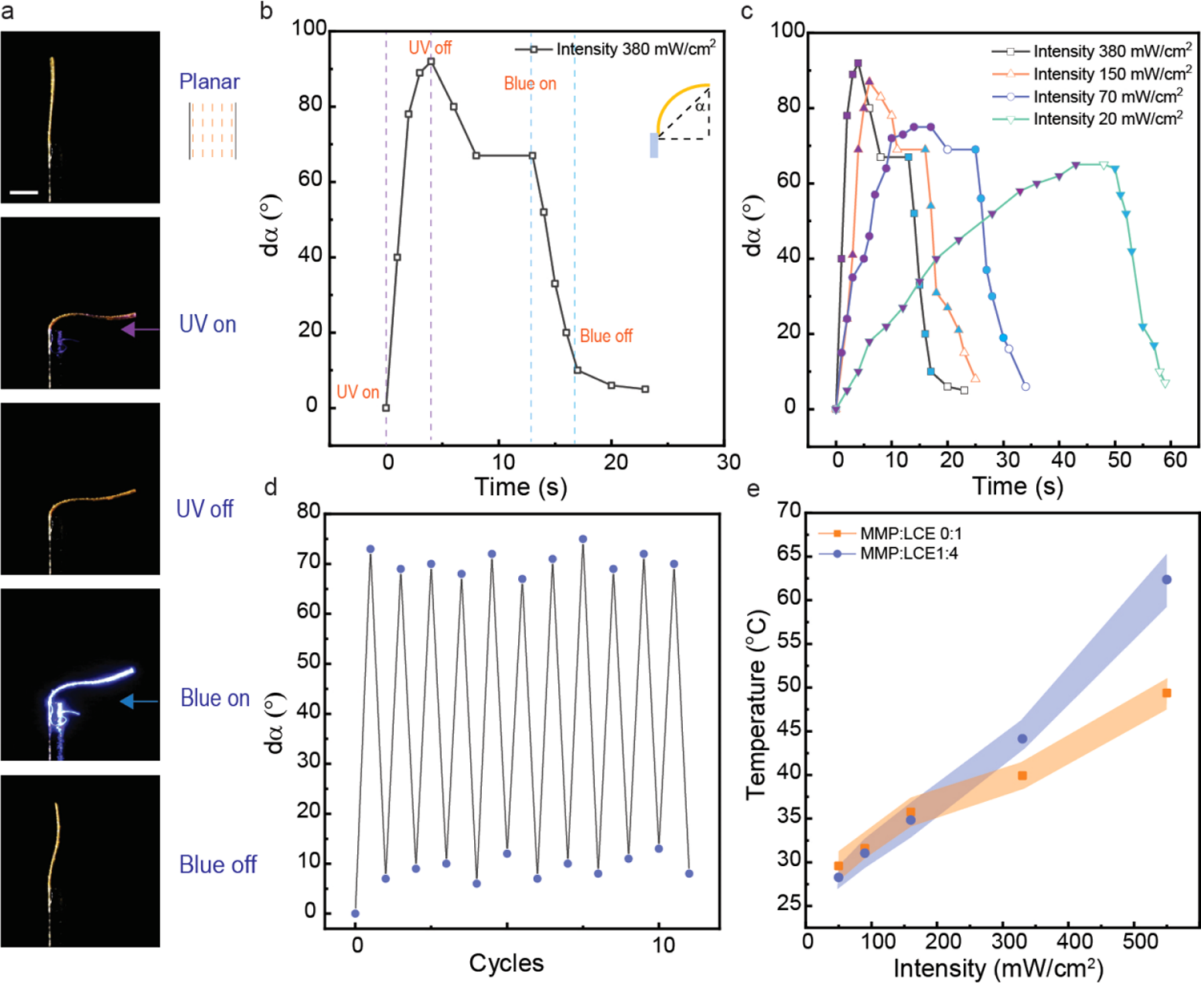
Photoactuation of the LCE–MMP composite. (a) Photographs illustrating the photochemical deformation process of the LCE-MMP composite (ρ = 1:4) under UV (365 nm, 120 mW cm^–2^) and blue (460 nm, 180 mW cm^–2^) light illumination. Scale bar: 3 mm. (b) Bending angle of the planar LCE strip upon irradiation with UV and blue light. (c) Bending angle of the LCE strip upon exposure to various UV light intensities and subsequent blue light irradiation. Filled symbols along the lines indicate the periods of light irradiation: purple-filled symbols correspond to the periods when UV light was turned on and blue-filled symbols correspond to the periods when blue light was turned on. Empty symbols denote periods when the light was turned off. (d) Cyclic photoinduced bending and unbending of LCE-MMP composite film upon alternating irradiation with 365 nm (95 mW cm^–2^) and 460 nm (180 mW cm^–2^). (e) Temperature recorded under different UV light intensities (365 nm) for the pristine LCE and the LCE-MMP composite (ρ = 1:4). Error bars indicate a standard deviation of *n* = 3 measurements.

For the photochemical actuation studies, we prepared planar-aligned strips of the LCE–MMP composite with dimensions of 5 × 1 × 0.05 mm^3^ and the director along the long axis of the strip. As illustrated in Figure 2a,b, upon exposure to UV light at 365 nm (120 mW cm^–2^), the LCE–MMP composite exhibits photochemically induced bending toward the light source due to *cis*-isomer gradient being formed through the thickness of the strip. After the cessation of UV exposure, the strip maintains the bent configuration. The slight unbending toward the original shape is due to slight photothermal heating under the experimental conditions used.^36,52^ Subsequent irradiation with blue light (460 nm, 180 mW cm^–2^) triggers reverse isomerization to the *trans* state, thus enabling the material to return to its initial shape. Figure 2c depicts the bending angle (dα) kinetics under different UV light intensities (20, 70, 150, and 380 mW cm^–2^) while keeping the UV dose approximately constant across all conditions and subsequent irradiation with blue light. Higher intensities lead to slightly more pronounced but significantly faster bending. The bending angle after cessation of UV irradiation is independent of the intensity used, indicating a similar *cis*-isomer population and gradient in all cases. We note that the presence of MMPs (ρ = 1:4) does not seem to hinder the bending process, but compared to the pristine LCE, the bending even seems slightly accelerated. When exploring this behavior in samples with higher MMP content (ρ = 1:2), we found that the bending angle reached is markedly lower (Figure S5). The bending process is fully reversible, as confirmed by 11 cycles of the alternate irradiation of the LCE–MMP composite (ρ = 1:4) with UV (365 nm, 95 mW cm^–2^, 30 s) and blue (460 nm, 180 mW cm^–2^, 30 s) light to observe the bending and unbending behavior (Figure 2d). The cycling stability underscores the material’s suitability for potential applications requiring durable and repeatable light-driven actuation cycles.

The photothermal response of the pristine LCE and the LCE–MMP composite across a range of light intensities from 50 to 550 mW cm^–2^ is presented in Figure 2e. At lower intensities (50 to 160 mW cm^–2^), the temperature increase is similar in both samples, indicating that MMPs do not markedly affect the photothermal conversion at these intensities. However, at intensities of 330 mW cm^–2^ and above, the presence of MMPs is associated with a more pronounced temperature rise. This observation indicates that in addition to azobenzenes, the MMPs serve as photothermal agents,^34^ leading to enhanced temperature increase under UV irradiation at higher light intensities.

**Figure 3:**
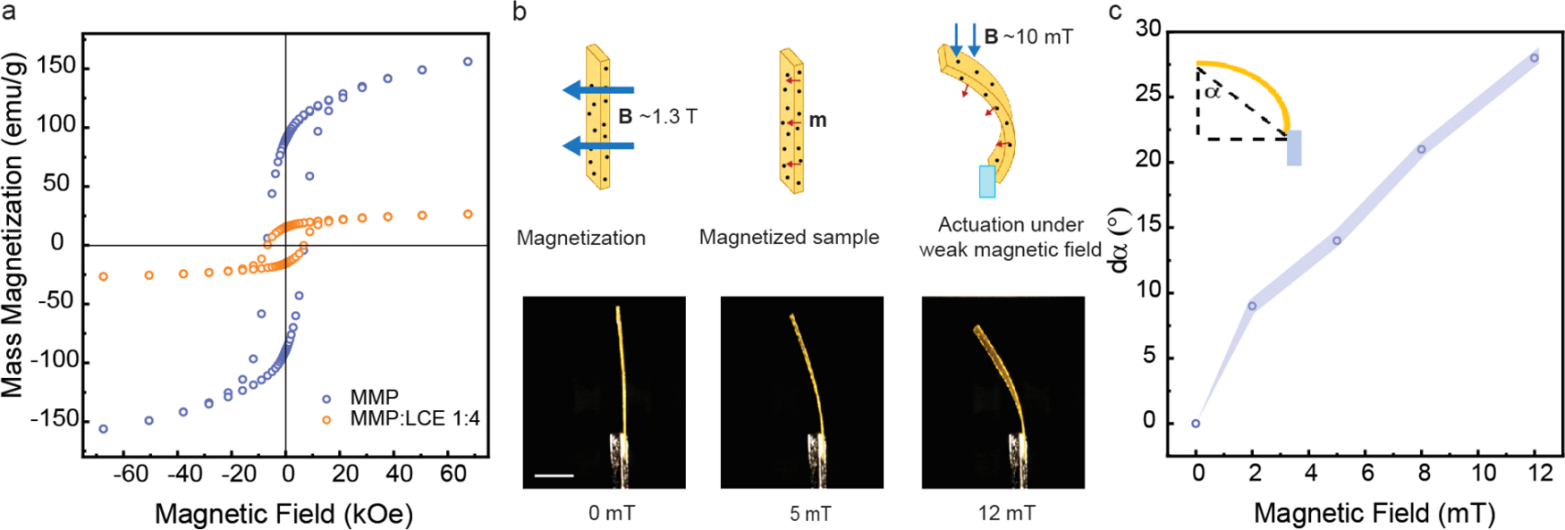
Magnetoresponse of the LCE–MMP composite. (a) Magnetic hysteresis curves of MMP and LCE-MMP composite. (b) Top: Schematic representation of the magnetization process. Bottom: Optical images of the LCE-MMP composite (ρ = 1:4) under applying the magnetic field. Scale bar: 5 mm. (c) Bending angle as a function of the magnetic field. Inset: indication of the strip bending angle for actuation measurements.

We next investigated the magneto-responsivity of the LCE–MMP composite (Figure 3). Magnetic hysteresis curves for pure MMPs and the LCE–MMP composite film were measured by using a vibrating sample magnetometer (VSM), as shown in Figure 3a. The VSM results indicate that the magnetoresponse of the composite film is proportional to the MMP mass ratio, decreasing from pure MMP due to the presence of the nonmagnetic LCE matrix. Despite the notable decrease, the preservation of magnetic functionality in the LCE–MMP composite still demonstrates a potent magnetic response, underscoring its potential for actuation in response to magnetic fields. This is evident from its remanence (the *y*-intercept) and coercivity (the *x*-intercept of the magnetization curve).

Figure 3b,c showcases the magneto-induced actuation of the LCE–MMP composite. The strip was initially magnetized along its surface normal using a strong magnetic field of 1.3 T, endowing the film with an orthogonal magnetic moment. Following this, applying a weaker magnetic field (in the mT range) led to the bending of the film, and the initial unbent state was retained once the field was turned off. The deformation observed under the magnetic field is attributable to a torque *τ* = *m* × *B* experienced by the LCE–MMP composite, where *m* is the magnetic moment and *B* is the external magnetic field, indicating that the torque is maximal when *m* and *B* are perpendicular to each other and zero when they are parallel. The torque acts to align the magnetic moment of the film with the magnetic field,^53^ causing the bending behavior observed in Figure 3b. The corresponding bending angles (dα) as a function of magnetic field strength are quantified in Figure 3c, depicting a gradual, approximately linear increase in bending angle with increasing magnetic field strength, consistent with the anticipated magneto-mechanical response.

Building on the foundational knowledge gained from the characterization and responsive behavior of the magneto-photoresponsive LCE composites, we proceeded to proof-of-concept demonstrations of underwater actuation. Compared to bimorph designs that achieve programmable deformation but may require supports, our LCE design maintains flexibility and robustness, essential for adaptive soft robotics.^54,55^

**Figure 4:**
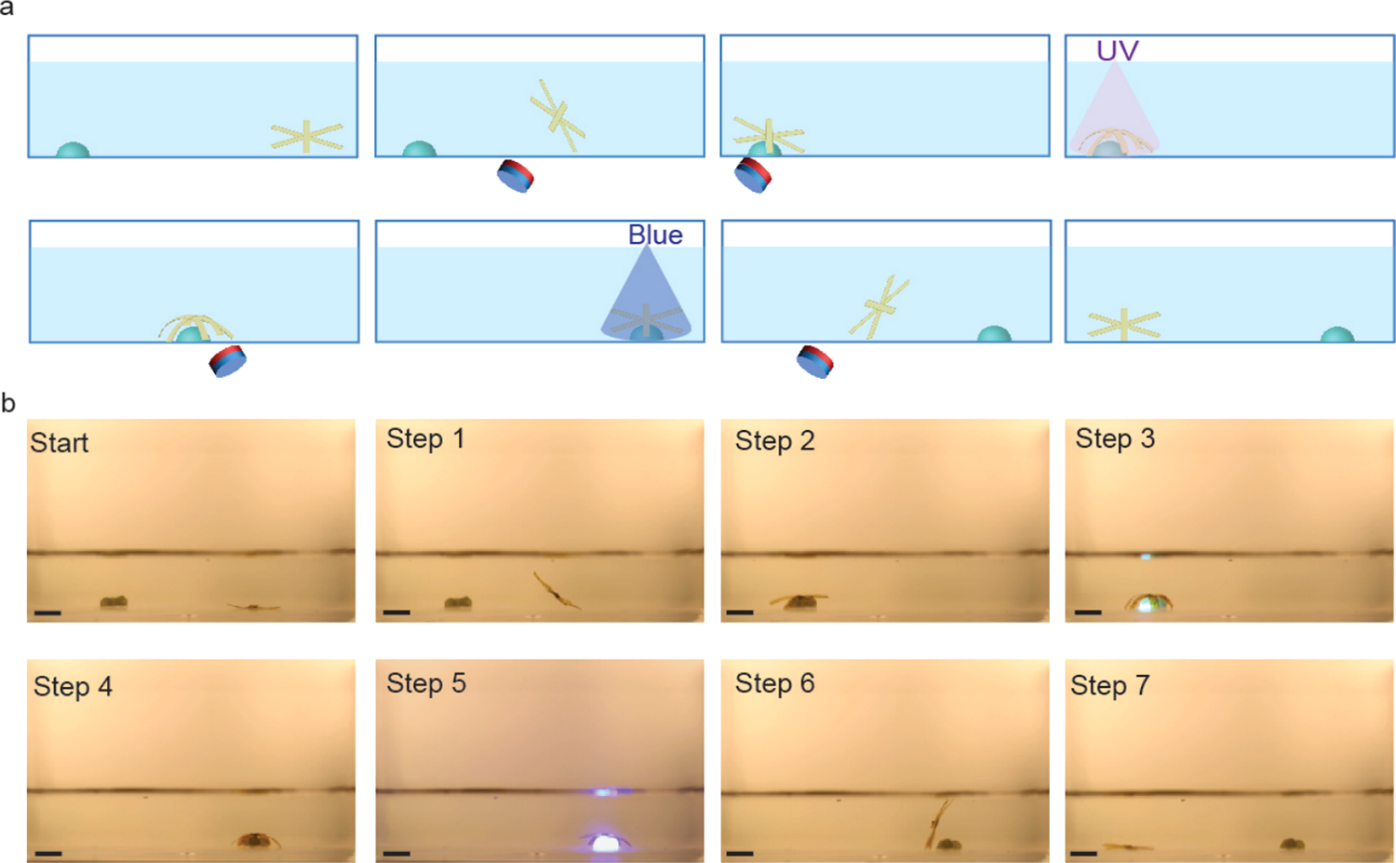
Magneto-photoinduced object translocation. (a) Schematic illustration of the sequential steps involved in the translocation process using a star-shaped LCE actuator. (b) Sequence of photographs showing the real-time actuation of the soft robot: at rest, bending upon UV exposure to hold an object, movement under the influence of a magnetic field, and release of the object upon blue light illumination. Scale bar: 5 mm.

Figure 4 showcases a star-shaped actuator crafted from three planar-aligned LCE–MMP strips glued together, exhibiting different functionalities in response to light and magnetic fields. UV exposure causes the arms of the actuator to bend in the direction of light irradiation due to the azobenzene-photoisomerization-induced photochemical actuation. This allows for the selective gripping and on-demand release of small-scale objects. For magnetic guidance, we utilized a small permanent magnet (disc magnet with a diameter of 12 mm and thickness of 3 mm) to remotely apply a magnetic field through manual translational and rotational movements beneath the water tank. This enabled the precise magnetically controlled transportation of the grasped object to a designated location. Upon arrival, exposure to blue light causes reverse isomerization, and the gripper releases the object at the specified spot. Subsequent application of the magnetic field can be used to retrieve the actuator while leaving the transported item in place (Movie S1, Supporting Information). Another demonstration of the actuator’s versatility is illustrated in Figure S6, which depicts a two-dimensional (2D) translocation maneuver, in which the actuator retrieves an object from the bottom of the tank and transports it to the water surface, where the object remains afloat due to surface tension.

**Figure 5:**
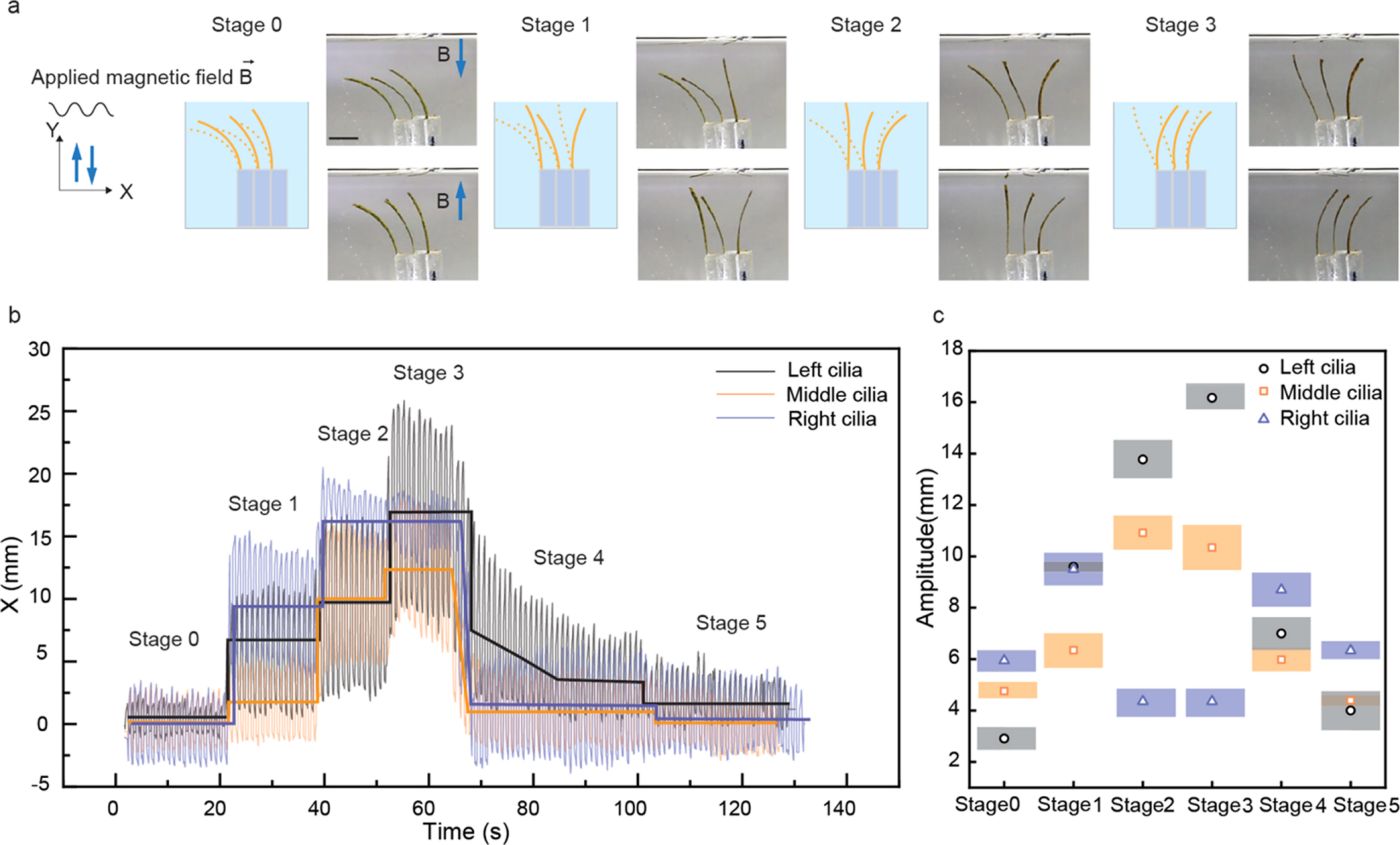
Photocontrolled oscillations of magneto-photoresponsive LCE cilia under oscillating magnetic field. (a) Schematic representation and sequential photographs depicting different actuation stages of the LCE cilia under sinusoidal magnetic field upon UV light exposure. The stages showcase the transition from initial oscillation without light (Stage 0) to photochemical control over the oscillation amplitudes (Stages 1 to 3). (b) Tip displacement in the *X* direction of the left, middle, and right cilia over time as they respond to the applied magnetic field and light stimuli, illustrating distinct oscillatory behaviors across the different stages of no light illumination (Stage 0), UV illumination (Stages 1 to 3), blue-light illumination (Stage 4), and retainment of the initial (Stage 5). (c) Amplitude quantification of the cilia oscillations during each stage, demonstrating orthogonal photocontrol over magneto-induced cilia oscillations. Scale bar: 1 cm.

The magneto-photoresponsive LCE also enables dynamic responses under sequential light illumination under an identical magnetic stimulus, as demonstrated in Figure 5 and Movie S2. We prepared a cilia-like structure from planar-aligned LCE strips that were magnetized along the surface normal. Three strips were fixed at one end on a glass substrate in a row with their tips free to move in response to external stimuli. When a sinusoidal magnetic field was applied in the vertical direction, the strips underwent oscillatory motion (Figure 5a, Stage 0), with a frequency dictated by the frequency of the applied magnetic field (Figure S7). The positional coordinates *X* and *Y* represent the horizontal and vertical positions of the cilia tips, respectively. Figure 5b narrates the light reconfigurability of the magnetically induced oscillation by tracking the horizontal (*X*) motion of the cilia tips. The solid lines overlaid on the oscillation data indicate the average position of the tips in the horizontal (*X*) direction over time, highlighting key changes in their motion pattern. Complementary to this, Figure 5c quantifies the amplitude of cilia oscillations during each stage. Oscillations of cilia in the vertical (*Y*) direction can be seen in Figure S8.

The oscillatory behavior can be divided into different stages. Stage 0 establishes the baseline, where no UV light is applied, allowing the sinusoidal magnetic field alone to set the cilia into an oscillatory motion. The slight differences in the initial oscillation amplitudes of each cilium are due to experimental constraints such as slightly different initial angles or positioning of the cilia and/or potential inhomogeneities of the magnetic field used. In Stage 1, UV light is turned on and manually positioned such that it mostly targets the rightmost cilium, causing it to bend toward the illumination direction. The bending alters the angle between the cilia’s long axis and the direction of the applied magnetic field, effectively changing the torque experienced by the cilia and hence the oscillation amplitude. We note that in addition to the changed magnetic torque, increased *cis*-population during irradiation also softens the LCE,^56^ which may potentially contribute to the changed oscillation amplitude. For the right cilium, this results in the maximum amplitude of oscillation observed during this stage, as depicted in Figure 5c.

In Stage 2, the UV illumination is manually repositioned such that the middle and left cilia are also exposed, resulting in greater overall bending of the cilia array. This modifies the magnetic torque and, consequently, the oscillation amplitudes. The middle cilium, in particular, experiences its maximum oscillation amplitude at this stage. The diminished oscillation amplitude of the right cilium at this stage is most likely explained by the nonhomogeneous magnetic field at the edge of the array, which results in a reduced effective magnetic torque. Stage 3 results in further modified magnetic torque and oscillation amplitudes, while in Stage 4, UV irradiation is ceased and blue light is used instead, triggering unbending toward the initial state. Stage 5 depicts the cilia after blue light exposure, resulting in oscillation amplitudes very similar to those in Stage 0. The small differences can be attributed to incomplete *cis–trans* photoisomerization that may have subtly softened the cilia, allowing a slightly greater response to the magnetic torque. This experiment demonstrates that photochemical bending allows light to serve as a control signal over the magnetic torque experienced by cilia in an aqueous environment. The outcome could be further improved by optimizing experimental conditions, such as achieving more precise photocontrol and ensuring a more uniform magnetic field.

Recent studies on hybrid composites^57,58^ highlight the importance of response stability under external stimuli. To evaluate the stability of the underwater actuation performance of our system, we conducted an experiment (Figure S9) monitoring the displacement of the cilium over a longer period (>2 min). This prolonged observation reveals consistent actuation response, with minimal variation in displacement amplitude, demonstrating the stability of the actuator under continuous operation.

## Conclusions

This study demonstrates that dual-responsive LCEs that combine magnetic and photochemical actuation capabilities provide advancements in underwater actuation controllability compared to LCEs responding to a single stimulus alone. The dual responsivity is achieved by incorporating azobenzene cross-links and magnetic microparticles into the LCE. The photoresponse and magnetoresponse are first separately characterized and then combined to demonstrate underwater object manipulation and photocontrollable oscillatory cilia motions, highlighting the effectiveness of the proposed dual-modality approach. The integration of the photochemical and magnetic response in a single material allows for precise, reversible control over the LCE shape-morphing. Separate functionalities can be induced by the two stimuli, or alternatively, one can be used as a control signal to fine-tune the response to the other. Our study demonstrates the power of combining these two remote actuation methods in the wireless control of robotic functions in responsive soft materials.

## Materials and Methods

### Materials

The LCE films are made by photopolymerization of a mixture containing 53 mol % of LC monomer 1,4-bis-[4-(6-acryloyloxyhexyloxy)benzoyloxy]-2-methylbenzene (RM82, Synthon Chemicals), 9 mol % of azo 4,4′-bis[6-(acryloyloxy)hexyloxy]azobenzene (Synthon Chemicals), 21 mol % chain extender dodecylamine (TCI), 17 mol % 6-Amino-1-hexanol, and 1.5 wt % of photoinitiator Bis(2,4,6-trimethylbenzoyl)-phenylphosphineoxide (Sigma-Aldrich). Hard magnetic microparticles (NdFeB, MQP-15–7, Magnequench, average diameter of 5 μm) were added to the photopolymerizable mixture at different concentrations prior to polymerization. All materials were used as received.

### LCE Film Preparation

To achieve planar alignment, two glass slides were spin-coated with a 5 wt % water solution of poly(vinyl alcohol) (PVA, 4000 rpm, 1 min). After being baked at 90 °C for 10 min, the slides were unidirectionally rubbed with a satin cloth. The cell assembly involved fixing two coated and rubbed substrates together with UV glue and spacing particles (Thermo Scientific, 50 μm) to set the cell thickness. The polymerizable mixtures (both with and without the MMPs) were infiltrated into the cell on a hot stage at 90 °C and then slowly cooled to the polymerization temperature (63 °C). The cell was kept in the oven for 24 h at 63 °C to allow the aza-Michael addition reaction for oligomerization to take place. The samples were then photopolymerized using a combination of 385 nm (200 mW cm^–2^) and 460 nm (150 mW cm^–2^) irradiation for 20 min (CoolLED pE-4000). We note that no external magnetic fields were used during the polymerization and that the magnetization was conducted separately. Finally, the cell was opened, and LCE strips were cut from the film by using a blade.

### Sample Characterization

Photographs and movies were captured using a Canon 5D Mark III camera equipped with a 100 mm lens. Thermal images were obtained with an infrared camera (FLIR T420BX) paired with a close-up 2× lens. An LED source (CoolLED pE-4000) was utilized for polymerization and actuation. Stress–strain curves were obtained by using a homemade tensile tester at a stretching speed of 0.05 mm/s. UV–vis absorption spectra and isomerization kinetics were recorded with an Agilent Cary 60 spectrophotometer. The alignment of the monodomain samples was characterized with a polarized optical microscope (Zeiss Axio Scope.A1) by imaging the samples with the director set to 0 and 45° angles between two crossed polarizers. VSM measurements were performed at room temperature by filling the VSM powder sample holder (Quantum Design, P125E) with the sample. The LCE-MMP sample film, measuring approximately 4.5 mm × 1.5 mm × 0.05 mm, was mounted vertically to the holder using 0.05 mm thick polyimide silicone adhesive (Kapton tape, Pro-power). Scanning electron microscopy (SEM, JEOL JSM-IT500) with an embedded energy dispersive spectroscopy system (EDS, JEOL) was used to observe cross-section of the LCE film. Differential scanning calorimetry (DSC) measurements were performed with a Netzsch DSC 214 Polyma instrument at a heating/cooling rate of 10 °C min^–1^.

### Magnetization and Magnetic Actuation

A permanent neodymium supermagnet (N45, 1.3 T) was used for magnetization. The LCE films (4.5 mm × 1.5 mm × 0.05 mm) were cut and fixed between two glass slides and then placed on top of the magnet to induce a magnetic moment perpendicular to the film’s surface. For magnetic actuation, magnetic fields (0 to 15 mT) were generated using two coaxially aligned coils, each with 1000 turns of wire. These coils were driven by a function generator (Tektronix Model AFG1022), which provided up to an 11 V voltage peak (*V*_p_) to control the magnetic field strength by adjusting the applied voltage. By applying a sinusoidal voltage through the function generator, a corresponding sinusoidal magnetic field was generated. During the experiments, the samples were positioned at the center between the coils at a distance of 1 cm from each.

## Supporting Information Available

Polarized absorption spectra to estimate the order parameter of LCE films; Cross-sectional SEM images; UV–vis absorption spectra, kinetic analysis; and switching cycles of LCE-MMP composites; bending angle of the LCE strip with different mass ratios of MMPs; real-time actuation sequences of a star-shaped LCE actuator; oscillation behavior of LCE cilia in response to different frequencies of applied magnetic fields and light stimuli (PDF)
Magneto-photoinduced object translocation (Movie S1) (MP4)
Photocontrolled oscillations of magneto-photoresponsive LCE cilia under an oscillating magnetic field (Movie S2) (MP4)
