# Supplementary material for: Magneto-Photochemically Responsive Liquid Crystal Elastomer for Underwater Actuation
Source: ACS Appl Mater Interfaces. 2025 Jan 9;17(3):5316–25. doi: 10.1021/acsami.4c14704 (PMC11758782; doi:10.1021/acsami.4c14704)
Supplement: Supplementary file 1 — am4c14704_si_001.pdf [file am4c14704_si_001.pdf]

Supporting Information

Magneto-photochemically responsive liquid crystal elastomer for underwater actuation

Yasaman Nemati[1], Qi Yang[1,2], Fereshteh Sohrabi[3], Jaakko V. I. Timonen[3], Carlos Sánchez-Somolinos[4,5], Mari Honkanen[6], Hao Zeng[1], Arri Priimagi[1]**

1. Faculty of Engineering and Natural Sciences, Tampere University, P.O. Box 541, FI-33101 Tampere, Finland.

2. Qingdao University of Science & Technology, Qingdao 266042, China

3. Department of Applied Physics, Aalto University School of Science, Puumiehenkuja 2, 02150, Espoo, Finland

4. Instituto de Nanociencia y Materiales de Aragón (INMA), CSIC-Universidad de Zaragoza, Departamento de Física de la Materia Condensada, Zaragoza 50009, Spain

5. Centro de Investigación Biomédica en Red de Bioingeniería, Biomateriales y Nanomedicina (CIBER-BBN), Instituto de Salud Carlos III, Madrid, 28029, Spain

6. Tampere Microscopy Center, Tampere University, P.O. Box 692, 33014 Tampere, Finland

Corresponding Authors

Arri Priimägi – arri.priimagi@tuni.fi

Hao Zeng – hao.zeng@tuni.fi

Supplementary Note 1:

To estimate the order parameter, we used a planar LCE film to measure the absorption spectra both parallel and perpendicular to the molecular director. The results of the polarized absorption spectra are presented in Figure S1. The order parameter, S, was calculated using the following formula:

$$S = \frac{A_{II} - A_{\perp}}{A_{II} + 2A_{\perp}} \quad (1)$$

where $A_{\parallel}$ and $A_{\perp}$ represent the absorbance measured with light polarized parallel and perpendicular to the liquid crystal (LC) alignment, respectively. For the calculations, we averaged data across the wavelength range of 475–500 nm and corrected the absorption spectra for any elevated baseline, ensuring the baseline was adjusted to zero.

Supporting Figures

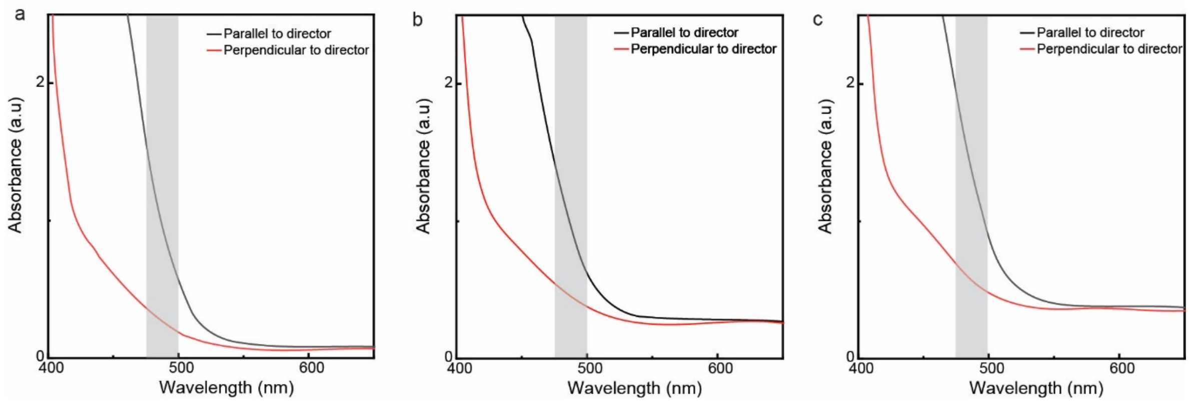

Figure S1. Polarized Absorption Spectra of Planar LCE Films at Varying MMP:LCE ratios. a) $\rho = 0:1$. b) $\rho = 1:4$. c) $\rho = 1:2$.

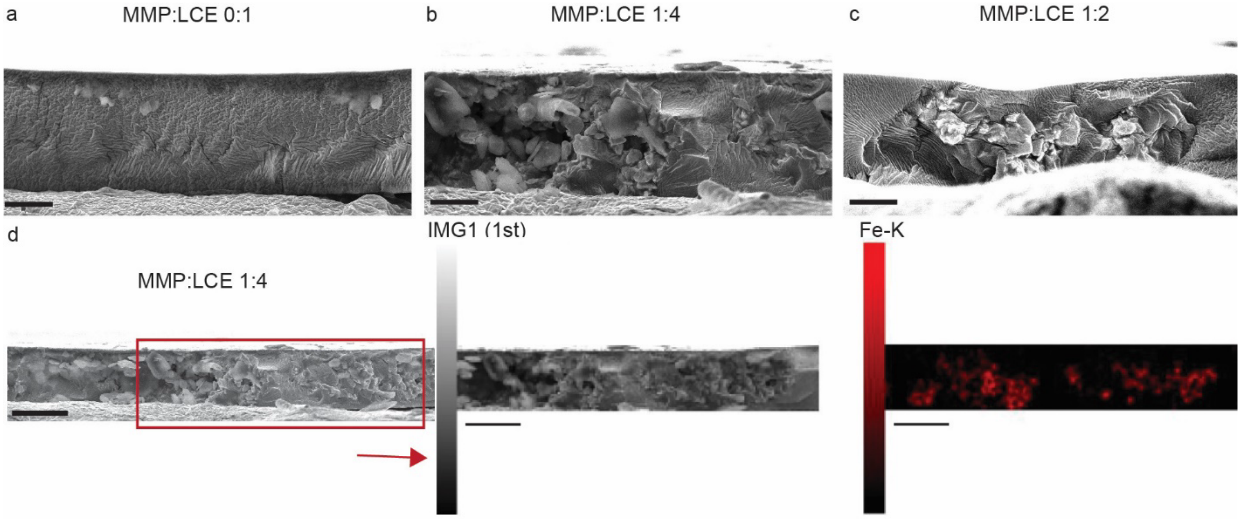

Figure S2. Cross-sectional SEM images of (a) pristine LCE and (b) $\rho = 1:4$ LCE-MMP composite, and (c) $\rho = 1:2$ LCE-MMP composite. (d) EDS elemental map of the $\rho = 1:4$ LCE-MMP composite, showing the iron distribution through the thickness of the film. Scale bars: 20 µm in (a-c), 50 µm in (d).

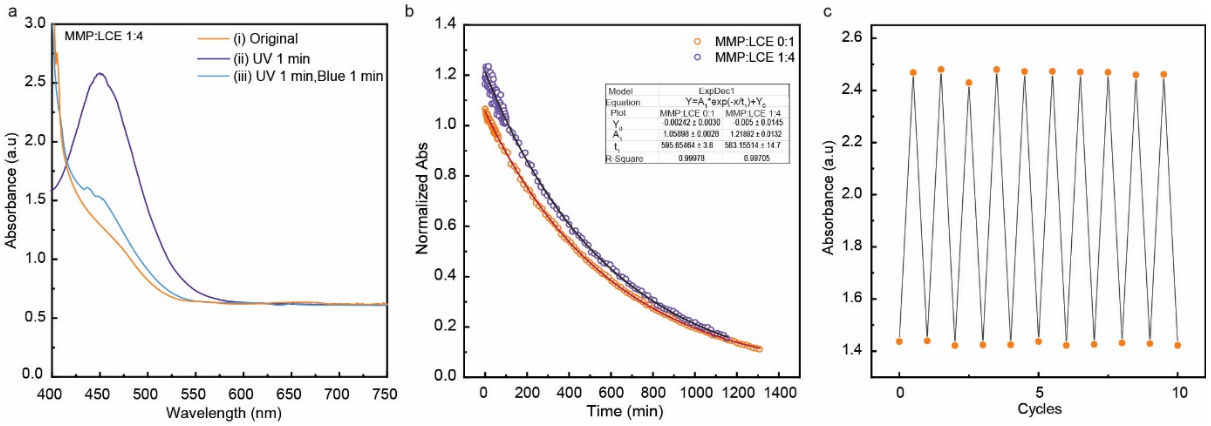

Figure S3. a) UV−vis absorption spectra of LCE–MMP composite. b) Kinetic analysis of the *cis*-lifetime of the LCE and the LCE–MMP composite. c) Switching cycles of the LCE–MMP composite film upon alternating irradiation with 365 nm (60 s, 155 mW cm^{-2}) and 460 nm (60 s, 180 mW cm^{-2}). The absorbance was monitored at 460 nm.

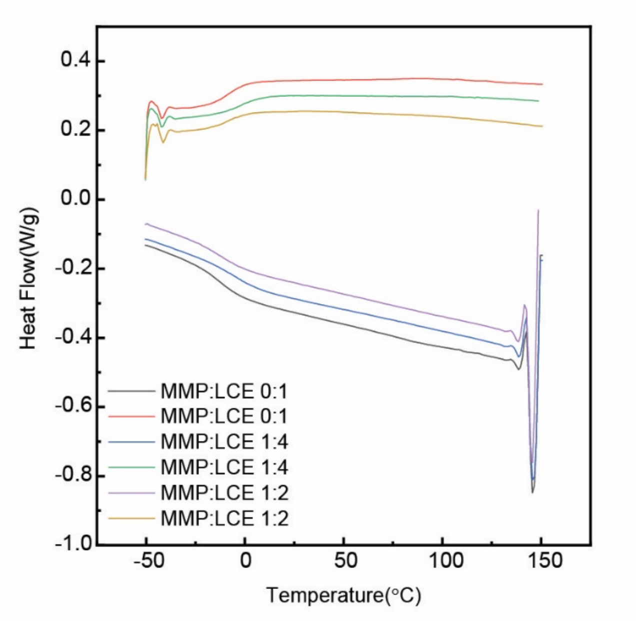

Figure S4. DSC curves of the pristine LCE and the LCE-MMP composites. The curves are from the second cooling and heating cycle. Heating and cooling speed: 10 °C min^{-1}.

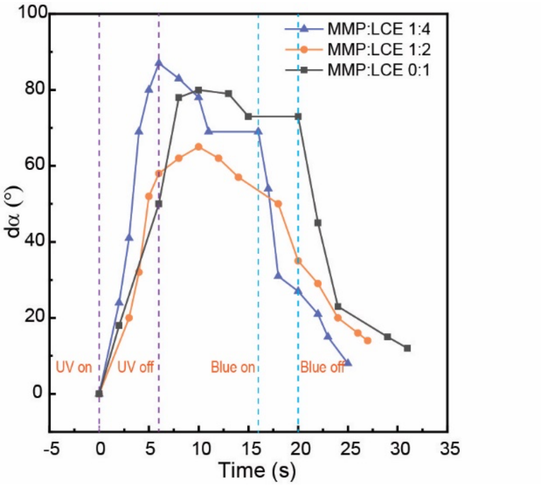

Figure S5. Bending angle of the LCE strip with different mass ratios of MMPs upon irradiation with UV (365 nm, 150 mW cm^{-2}) and blue (460 nm, 180 mW cm^{-2}) light. Vertical dashed lines show the timeline of irradiation for $\rho = 1{:}4$.

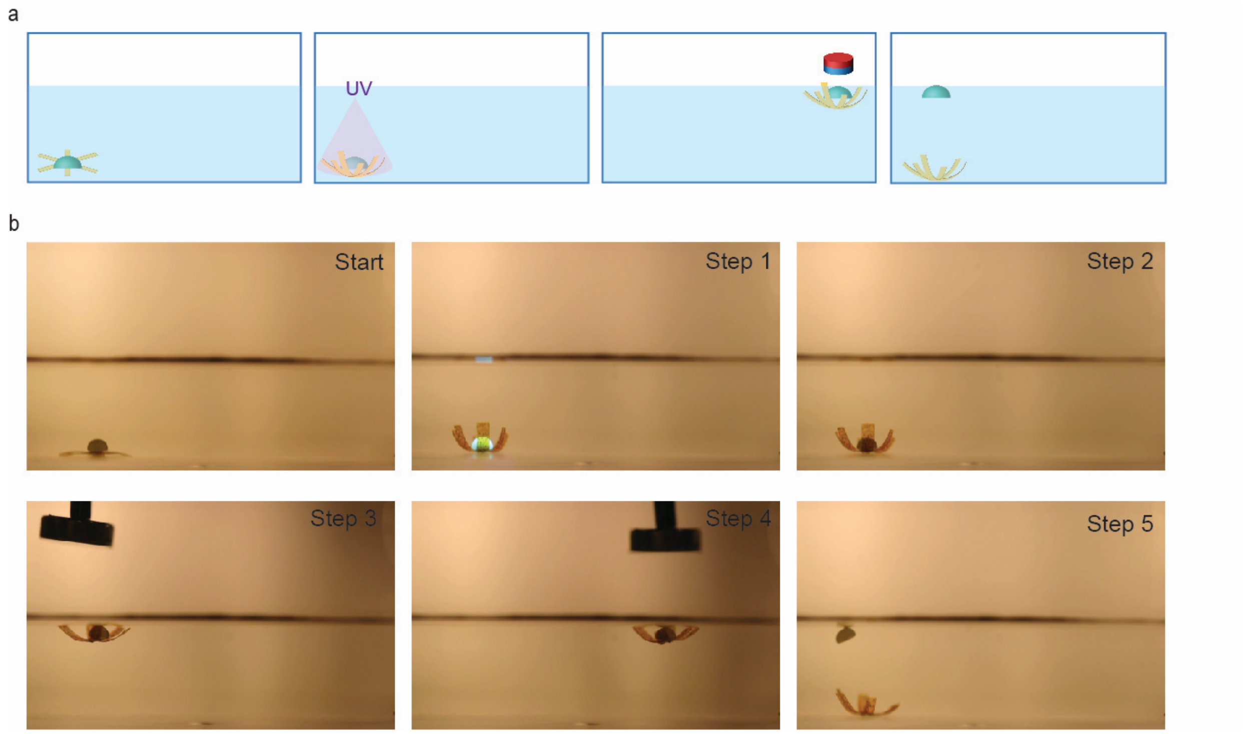

Figure S6. a) Schematic illustration of the sequential steps involved in the translocation process using a star-shaped LCE actuator. (b) Sequence of photographs showing the real-time actuation of the soft robot: at rest, bending upon UV exposure to hold an object, movement under the influence of a magnetic field and bringing to surface, and release of the object upon removal of magnetic field.

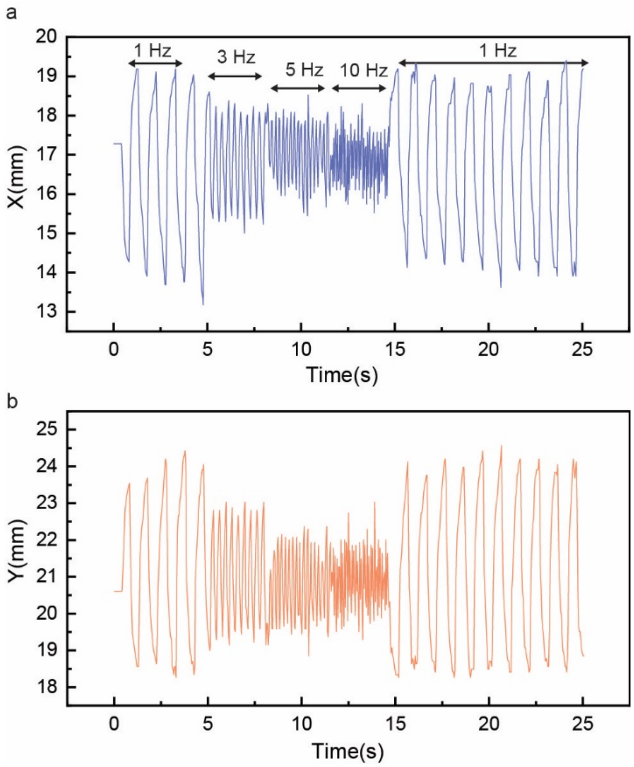

Figure S7. Oscillation behavior of an LCE cilium in X direction (a) and Y direction (b) upon different frequencies of the applied magnetic field.

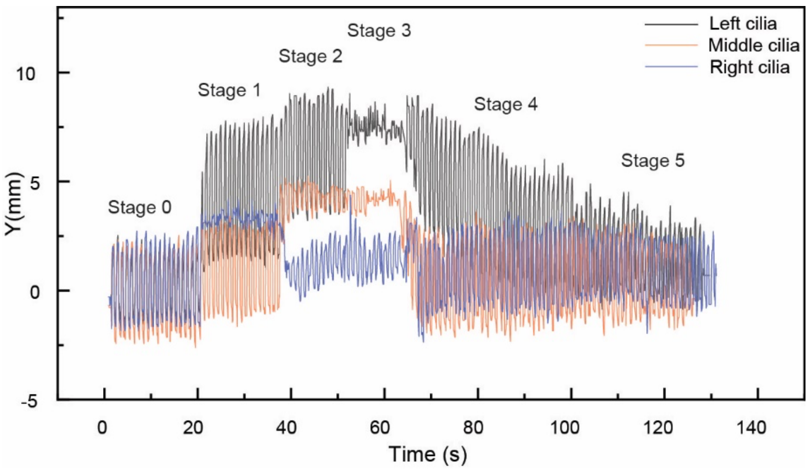

Figure S8. Vertical displacement (Y) of the left, middle, and right cilia over time as they respond to the applied sinusoidal magnetic field and light stimuli.

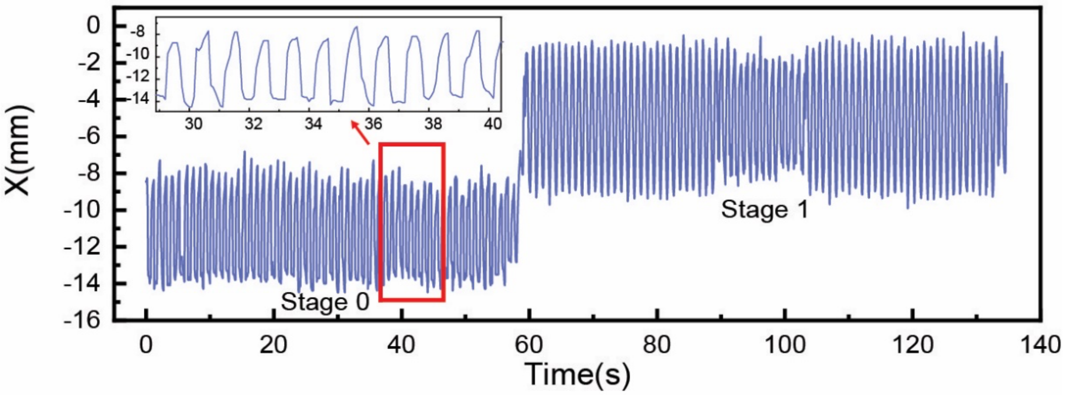

Figure S9. Tip displacement of the cilium in the X direction over extended time period in response to sinusoidal magnetic field in the dark (Stage 0) and under UV illumination (Stage 1). Inset: magnified view of the oscillatory behavior.
